# Sexual education and body estimation among women in early adulthood - Polish experience

**DOI:** 10.1192/j.eurpsy.2023.1343

**Published:** 2023-07-19

**Authors:** M. Gawrych, M. Kaźmierska, M. Em-Kamieniec

**Affiliations:** Institute of Psychology, The Maria Grzegorzewska University, Warsaw, Poland

## Abstract

**Introduction:**

Chronic sexual health problems often emerge in young adulthood, when adults begin to build the close relationship and develop lifelong sexual health behaviors. The associations between body estimation and sex education, especially in women group, is not well known in Poland.

**Objectives:**

The aim of the research was to check a relationship between body estimation and (1) taking part in formal sex education classes in the past and (2) subjective rating of one’s knowledge in the field of sexuality among women in early adulthood.

**Methods:**

We examined 159 women between 18 and 35 years. We used a survey, consisted of respondents’ particulars and Body Esteem Scale (Franzoi, Shields, 1984) including 35 items related to body. BES consists of three subscales for women (Sexual attractiveness, Weight concern, Physical condition) and three subscales for men (Physical attractiveness, Upper body strength and Physical condition). Respondents had to pick one of the answers on a five-point scale, where 1 meant „strong negative feelings” and 5 meant „strong positive feelings”. The study was conducted online, via MS Forms platform.

**Results:**

There was a statistically significant relationship between taking part in formal sex education classes in the past and perception of physical condition among women. The analysis also proved the relationship between subjective rating of one’s knowledge in the field of sexuality and rating of one’s sexual attractiveness and perception of these parts of body that can be changed through physical exercises or dieting. Additionally, the research showed a statistically significant relationship between rating of one’s knowledge in the field of sexuality and general estimation of one’s body.

**Conclusions:**

By leading reliable sexual education one may affect better attitude to bodies among young adults, who are in a sensitive phase for building stable relationships with other people. Simultaneously one may improve their mental, physical and social well-being.

**Disclosure of Interest:**

None Declared

